# Chamazulene Attenuates ROS Levels in Bovine Aortic Endothelial Cells Exposed to High Glucose Concentrations and Hydrogen Peroxide

**DOI:** 10.3389/fphys.2018.00246

**Published:** 2018-03-20

**Authors:** Giulia Querio, Susanna Antoniotti, Federica Foglietta, Cinzia M. Bertea, Roberto Canaparo, Maria P. Gallo, Renzo Levi

**Affiliations:** ^1^Department of Life Sciences and Systems Biology, University of Turin, Turin, Italy; ^2^Department of Drug Science and Technology, University of Turin, Turin, Italy

**Keywords:** Chamazulene, oxidative stress, H_2_O_2_, glucose, bovine aortic endothelial cells, flow cytometry, confocal microscopy, ROS

## Abstract

Endothelial cells surround the lumen of blood vessels and modulate many physiological processes, including vascular tone, blood fluidity, inflammation, immunity and neovascularization. Many pathological conditions, including hyperglycemia, may alter endothelial function through oxidative stress, leading to impaired nitric oxide bioavailability and to the onset of an inflammatory state. As widely shown in the last decade, dietary intervention could represent a good strategy to control endothelial dysfunction and atherosclerosis. In particular, extensive research in the field of antioxidant natural derivatives has been conducted. In this study, we evaluated the capability of Chamazulene (Cham), an azulene compound from chamomile essential oil, to attenuate ROS levels in bovine aortic endothelial cells (BAECs) stressed with either high glucose or H_2_O_2_. Cell viability at different concentrations of Cham was evaluated through the WST-1 assay, while ROS production acutely induced by High Glucose (HG, 4.5 g/L) treatment or H_2_O_2_ (0.5 mM) for 3 h, was quantified with 2′-7′-Dichlorofluorescein diacetate (DCFH-DA) probe using confocal microscopy and flow cytometry. Our results showed a reduction in ROS produced after simultaneous treatment with High Glucose or H_2_O_2_ and Cham, thus suggesting an *in vitro* antioxidant activity of the compound. On the whole, this study shows for the first time the potential role of Cham as a scavenging molecule, suggesting its possible use to prevent the rise of endothelial ROS levels and the consequent vascular damage.

## Introduction

**Figure 1:**
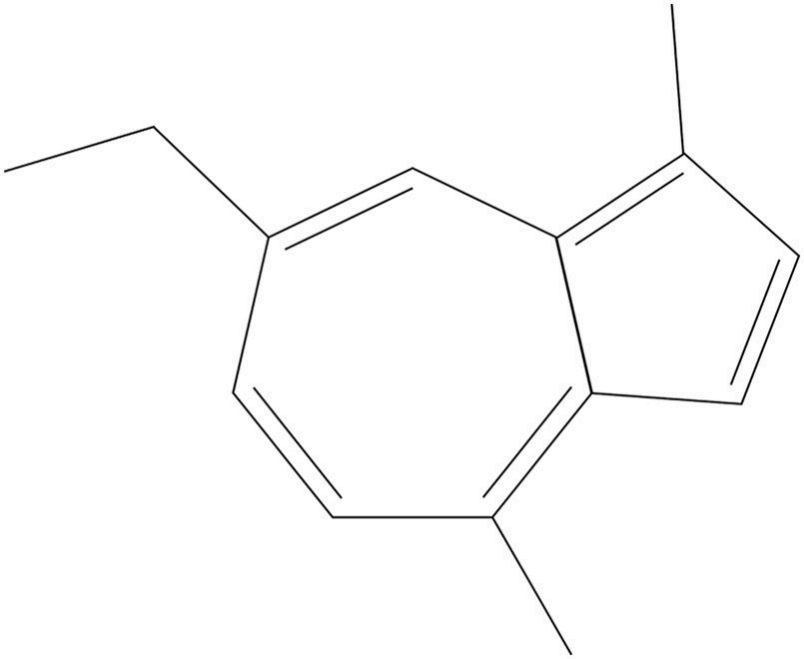
Chemical structure of Chamazulene, C.A.S. n 529-05-5.

In recent years studies on free radicals, as reactive oxygen species (ROS) and reactive nitrogen species (RNS), and their role in mediating different functions in our organism are increasing. Free radicals, ROS and RNS normally produced in living cells, can increase due to external sources, such as X-rays, air pollutants or chemical compounds, or can be endogenously produced by essential enzymatic or non-enzymatic processes (Lobo et al., 2010). These molecules are involved in oxidation-reduction (redox) reactions. Indeed, free radicals are characterized by an unpaired electron that makes these molecules highly unstable and able to act both as oxidants or reductants. Such characteristics are fundamental in the regulation of different cellular functions, collectively indicated as “redox signaling” (Sies, 2015), and underline the role of these molecules both in physiological and pathological conditions. For example, ROS produced by phagocytic cells are fundamental in the first defense against infections (Finkel and Holbrook, 2000), but their uncontrolled rise with the consequent generation of a redox state, called the oxidative stress status, can be deleterious for cellular structures, like DNA, proteins and lipids, with the consequent modification of their function (Espinosa-Diez et al., 2015). Possible generation of systemic long term complications, such as chronic inflammation, endothelial dysfunction, atherosclerosis and cancer, can be related to this condition (Sies, 2015). To control the production of free radicals, ROS and RNS, animal cells use different systems generically called antioxidants, molecules able to donate an electron to free radicals, neutralizing them and confining cell damage. Antioxidants are classified in enzymatic and non-enzymatic molecules. Among enzymatic there are superoxide dismutase (SOD), catalase (CAT), glutathione peroxidase (GSH-Px), thioredoxins (TRX), peroxiredoxins (PRX), glutathione transferase (GST). Examples of non-enzymatic antioxidants are glutathione (GSH), ferritin, transferrin, uric acid, coenzyme Q (Birben et al., 2012). The importance of diet antioxidants that can modulate and sustain endogenous defenses is emerging: carotenoids, vitamin C, vitamin E, omega-3 fatty acids, β-glucans and polyphenols, present in food, can be used by the organisms to reinforce their antioxidant response (Kofuji et al., 2012; Pisoschi and Pop, 2015). Furthermore, in last years, the role of officinal plants as cellular endogenous defense enhancers or free radical *scavengers* emerged and the possible use of plant extracts, essential oils or isolated molecules of traditional relevance as antioxidants is nowadays strongly investigated (Agatonovic-Kustrin et al., 2015; Pisoschi and Pop, 2015). Among officinal plants, chamomile (*Matricaria chamomilla* L.) demonstrated several beneficial properties in cell cultures and in *in vivo* studies (McKay and Blumberg, 2006). In fact, chamomile infusions and extracts showed anti-inflammatory, anti-microbial, hypocholesterolemic and anti-genotoxic effects (Petronilho et al., 2012). Different studies showed that secondary metabolites, in particular terpenoids and flavonoids, isolated from chamomile, are able to neutralize the propagation of radical chains thank to their molecular structure, underlining the possibility to classify these molecules as natural antioxidants (Singh et al., 2010). Among bioactive compounds present in chamomile essential oil, Chamazulene (Cham), a sesquiterpene derived from matricine (Singh et al., 2010), has been proposed as a free radical scavenger. Results obtained with antioxidant assays, like that based on the 2,2′-azino-bis-3-ethylbenzthiazoline-6-sulphonic acid (ABTS) radical (Capuzzo et al., 2014; Agatonovic-Kustrin et al., 2015), suggested the possible antioxidant role of this molecule in a cell model of acute or chronic oxidative stress. Although available data in literature suggest Cham as a radical *scavenger*, there are no studies on its possible effect in a cell model of oxidative stress. In fact its chemical nature suggests its passage through the cell membrane and the possible interaction with radical species (Figure 1). Oxidative stress cell models can be determined in different ways depending on cell type and on their susceptibility to specific stressors that can cause pathophysiological conditions. For example, the diabetic state is characterized by endothelial dysfunction, induced by different stressors like high glucose concentrations (Zhou et al., 2015), which cause diminished production of nitric oxide, and, as a consequence, an imbalance in endothelium-derived relaxing and contracting factors, up-regulation of adhesion molecules, increased chemokine secretion, leukocyte adherence and cell permeability, low-density lipoprotein oxidation, platelet activation and vascular smooth muscle cell proliferation and migration (Hadi et al., 2005). The aim of this study was to evaluate the antioxidant properties of Cham on bovine aortic endothelial cells (BAECs) acutely treated with two different oxidative stressors already proposed in other studies (Zhou et al., 2015; Nadeev et al., 2016): High Glucose (HG, 4.5 g/L) concentrations or hydrogen peroxide (H_2_O_2_, 0.5 mM).

## Materials and methods

### Chemicals

Chamazulene purification from chamomile (*Matricaria chamomilla* L.) essential oil was performed according to Capuzzo et al. (2014). The compound used was solubilized in absolute ethanol (Sigma Aldrich Saint Louis, MO, USA) at the final concentration of 10 mg/ml. Unless otherwise specified, all reagents for cell culture and experiments were purchased from Sigma-Aldrich.

### Cell culture

Bovine aortic endothelial cells-1 (BAECs, ECACC, Salisbury, UK) were maintained in Dulbecco's modified eagle medium (DMEM) 1 g/L glucose supplemented with 10% FBS, 2 mM L-Glutamine and 50 μg/ml Gentamycin, incubated at 37°C in a humidified atmosphere containing 5% CO_2_. High glucose (HG) treatment was performed using DMEM 4.5 g/L glucose supplemented with 10% FBS, 2 mM L-Glutamine and 50 μg/ml Gentamycin. H_2_O_2_ treatment was performed at the dilution of 0.5 mM in DMEM 1 g/L glucose. BAECs were used from passages 3 to 6.

### Assessment of cell viability after chamazulene treatment

Cell proliferation reagent WST-1 (Roche Applied Science, Mannheim, Germany) based on the cleavage of a tetrazolium salt into a formazan product by living cell enzymes, in particular mitochondrial dehydrogenases, was used to assess cell viability. BAECs (1.6 × 10^3^ cells/100 μl/well) were seeded into a 96-well plate in culture medium and incubated at 37°C for 24 h. Following incubation, cells were treated with increasing concentrations (10, 25, 100, 250 μg/ml) of Cham or its solvent, ethanol, at the same dilutions (1:1000; 1:400; 1:100; 1:40) for 3 h. WST-1 solution (1:10) was added 2 h before the end of the treatment. The absorbance at 450 nm was determined by a microplate reader (Microplate Reader, Bio-Rad, model 550). The effect of Cham on cell viability was calculated from the absorbance of soluble formazan dye generated by living cells and the results were expressed as percentage of cell viability compared to control, fixed at 100%.

### Determination of the EC_50_ of chamazulene

Half maximal effective concentration (EC_50_) of Cham was obtained studying the effect of the compound at different concentrations (10, 25, 100, 250 μg/ml) for 3 h by means of the WST-1 Assay; from these data, the EC_50_ was calculated using the software CalcuSyn 2.11 (Biosoft, Cambridge, UK).

### ROS measurement with confocal microscopy

BAECs production of ROS was assessed with confocal microscopy using 2′-7′-Dichlorofluorescein diacetate probe (DCFH-DA, Sigma). BAECs were seeded (4.8 × 10^4^ cells/ml) on uncoated glass bottom dishes of 35 mm diameter (MatTeck Corporation, Ashland, MA, USA) in DMEM 1 g/L glucose and incubated at 37°C for 24 h. Following incubation cells were treated with Cham 25 μg/ml, HG, H_2_O_2_ or simultaneously treated with Cham 25 μg/ml plus HG and Cham 25 μg/ml plus H_2_O_2_ for 3h; a control condition with ethanol 1:400, correspondent to that present in the 25 μg/ml Cham solution, was added to evaluate its effect alone on the cells. DCFH-DA solution (1 μl/ml) was added to each dish 30 min prior the end of the treatment, then cells were washed three times with PBS containing Ca^2+^ and Mg^2+^ to avoid cells loss. Fluorescence at 488 nm was determined with confocal microscopy (magnification 60x). Quantitative ROS production was calculated with the definition and measurement of Regions Of Interest (ROIs) using the software ImageJ (Rasband, W. S., ImageJ, U. S. National Institutes of Health, Bethesda, Maryland, USA, https://imagej.nih.gov/ij/, 1997-2017) and expressed as relative Medium Fluorescence Index (MFI) compared to control, fixed at 1.

### ROS measurement with flow cytometry

BAECs ROS production was assessed with flow cytometry (C6 Accuri, BD Bioscience) using DCFH-DA probe. BAECs were seeded into a 6-well plate (7 × 10^4^ cells/well) in culture medium and incubated at 37°C for 24 h. Following incubation, cells were treated for 3 h with Cham 25 μg/ml, HG, H_2_O_2_ or simultaneously treated with Cham 25 μg/ml plus HG and Cham 25 μg/ml plus H_2_O_2_; a control condition with ethanol 1:400 was added to evaluate its role in mediating the effect of Chamazulene. DCFH-DA solution (1 μl/ml) was added to each well 30 min prior the determination of the fluorescence. A total of 10,000 events were considered and ROS levels were recorded at 5 min. Quantitative ROS production was calculated as relative Medium Fluorescence Index (MFI) compared to control, fixed at 1.

### Statistical analysis

All data were expressed as mean ± Standard Deviations of the mean. For differences between mean values Bonferroni's multiple comparisons test was performed. Differences with *P* < 0.05 were regarded as statistically significant.

## Results

### BAECs viability after exposure to different concentrations of chamazulene

**Figure 2:**
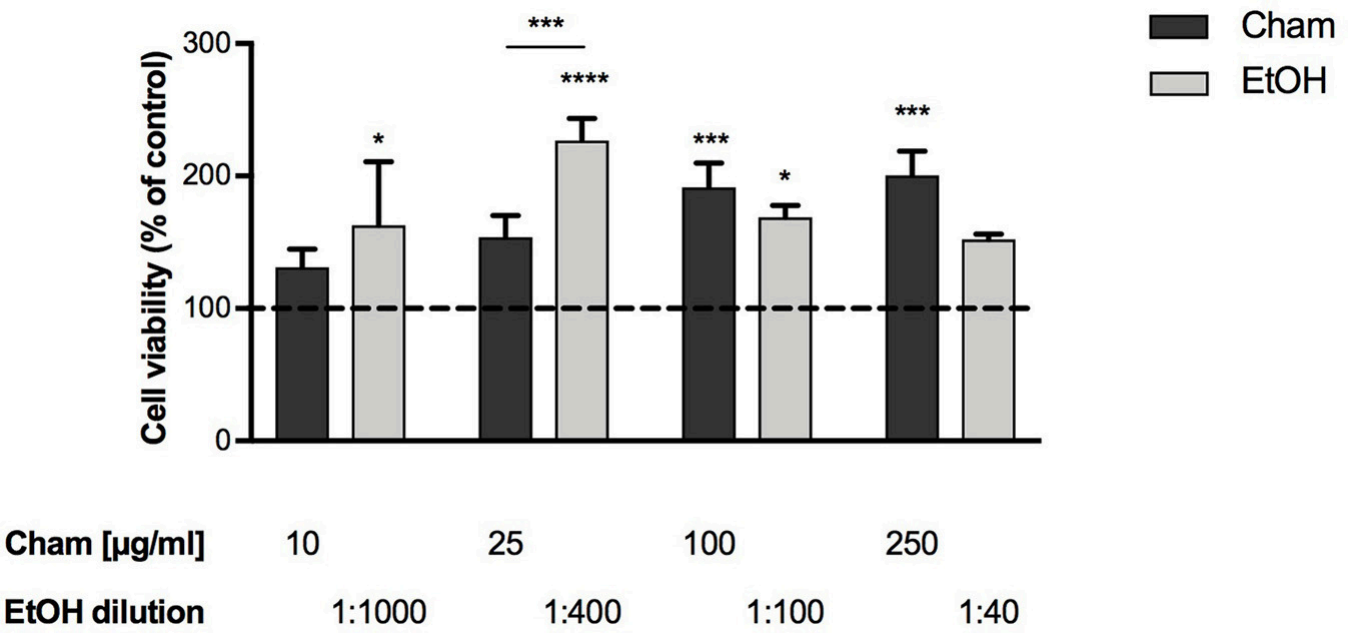
Effect of Chamazulene on BAECs at 3 h of treatment, as assessed by the WST-1 Assay. None of the concentrations used was toxic for BAECs. Cell viability was not affected by ethanol (light gray bars) used at the same amount present in each Cham treatment. Data shown are mean ± SD of three independent experiments and are expressed as percentage toward control (Data are presented from the lowest to the highest concentration used. Cham: 131.08 ± 13.91; 153.81 ± 16.50; 191.56 ± 18.18; 200.36 ± 18.38. EtOH: 162.68 ± 48.13; 226.67 ± 16.97; 168.90 ± 9.02; 152.22 ± 3.94); ^*^P < 0.05, ^***^P < 0.001, ^****^P < 0.0001.

To assess Cham toxicity BAECs were exposed to different concentrations of the compound (10, 25, 100, 250 μg/ml) for 3 h; its effects were evaluated by means of the WST-1 assay. None of the concentrations tested was toxic at 3 h; and, as shown in Figure 2, cells viability was not affected by ethanol at any of the concentrations used, corresponding to the amount of the solvent present in each Cham treatment.

These data were used to calculate the EC_50_ of Cham with the software CalcuSyn and, as confirmed in Figure 2, 25 μg/ml increased cells viability at 50% in 3 h and represented the EC_50_ dose of the compound in these experiments. This concentration was used in this preliminary evaluation of the antioxidant activity of Cham in cells.

### Chamazulene and ROS levels after HG treatment

**Figure 3:**
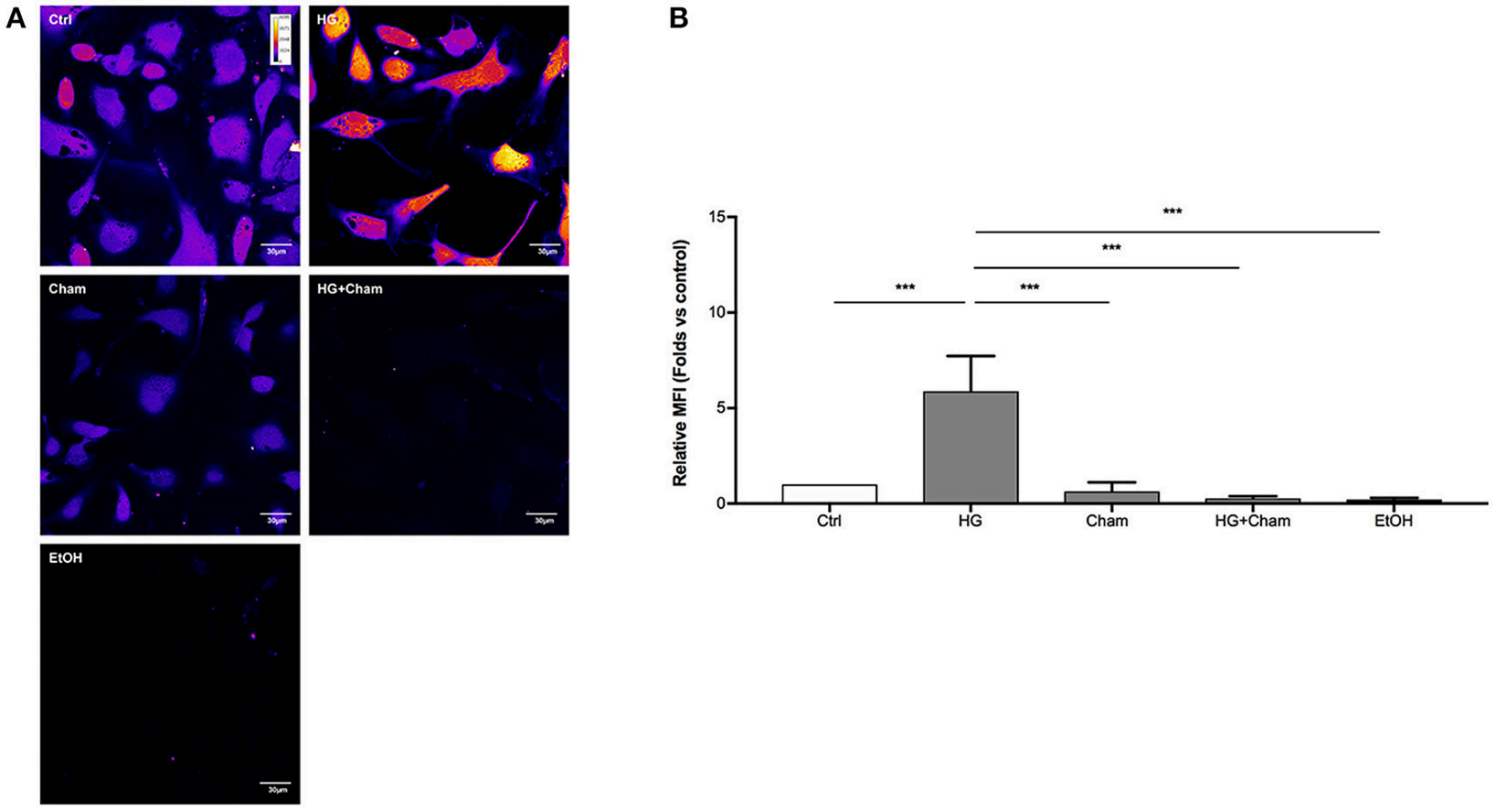
Chamazulene and ROS levels in confocal microscopy after HG treatment. **(A)** Images obtained with confocal microscopy and DCFH-DA probe (magnification 60x). Fluorescence intensity is represented in pseudo color scale (“fire” in ImageJ software): HG treatment for 3 h induced augmented ROS levels as underlined by the changing color of cells compared to control and it was balanced by the simultaneous treatment with Cham. **(B)** Histograms illustrate the relative MFI toward control derived from images analysis and show augmented ROS levels when cells are treated with HG and their decrease in a simultaneous treatment with Cham. Cham effect is not affected by ethanol. Data shown are mean±SD of three independent experiments (HG: 5.89 ± 1.84; Cham: 0.65 ± 0.47; HG + Cham: 0.29 ± 0.10; EtOH: 0.22 ± 0.09); ^***^P < 0.001.

**Figure 4:**
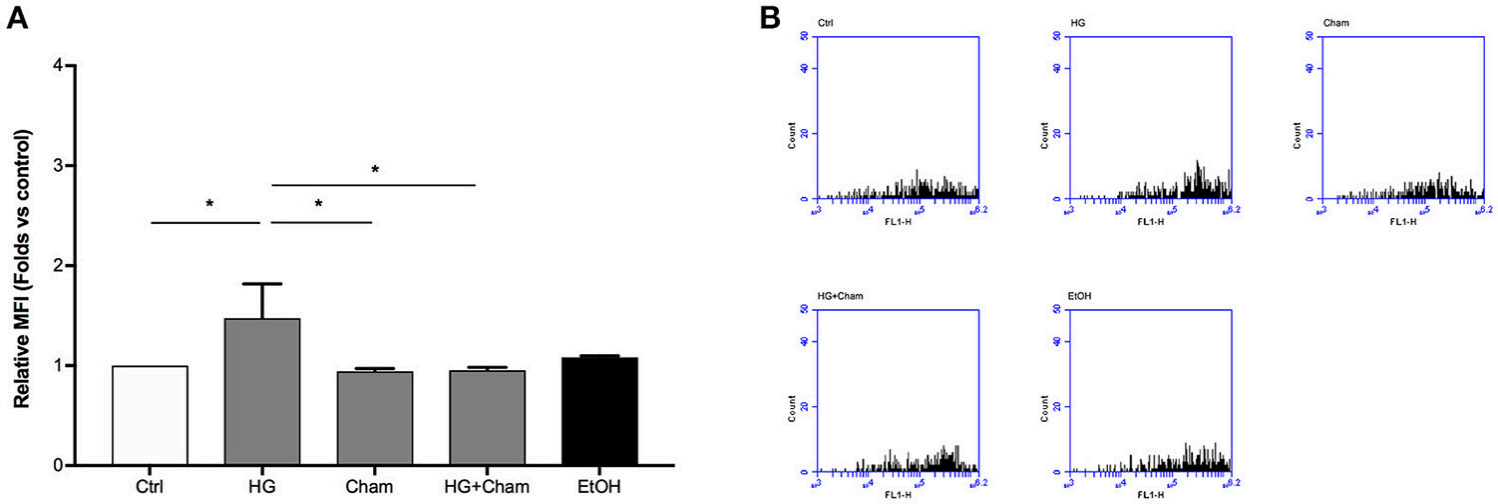
Chamazulene and ROS levels in flow cytometry after HG treatment. **(A)** Histograms illustrate the relative MFI toward control derived from flow cytometry and show ROS levels when cells are treated with HG and their decrease in a simultaneous treatment with Cham and HG. Cham effect is not affected by ethanol. Data shown are mean ± SD of three independent experiments (HG: 1.47 ± 0.35; Cham: 0.94 ± 0.03; HG + Cham: 0.96 ± 0.02; EtOH: 1.08 ± 0.02); ^*^P < 0.05 **(B)** Representative graphs of flow cytometry in which is illustrated the variation in fluorescence in different treatments.

ROS production in BAECs after HG treatment was at first evaluated by confocal microscopy using the DCFH-DA probe (Figures 3A,B). Cells were treated for 3 h with HG, Cham 25 μg/ml or simultaneously with both. High Glucose treatment for 3 h induced augmented ROS levels that was balanced by the simultaneous addition of Cham. A control condition with ethanol 1:400, corresponding to the amount of solvent added to cells with the 25 μg/ml Cham treatment, had no effect on BAECs. Results of confocal microscopy experiments were confirmed by flow cytometry: high glucose treatment induced a significant ROS increase in BAECs, as compared to cells maintained in low glucose medium; Cham added together with HG was able to attenuate the effect of HG while nor Cham nor EtOH alone did not have any effect on ROS production (Figures 4A,B).

### Chamazulene and ROS levels after H_2_O_2_ treatment

**Figure 5:**
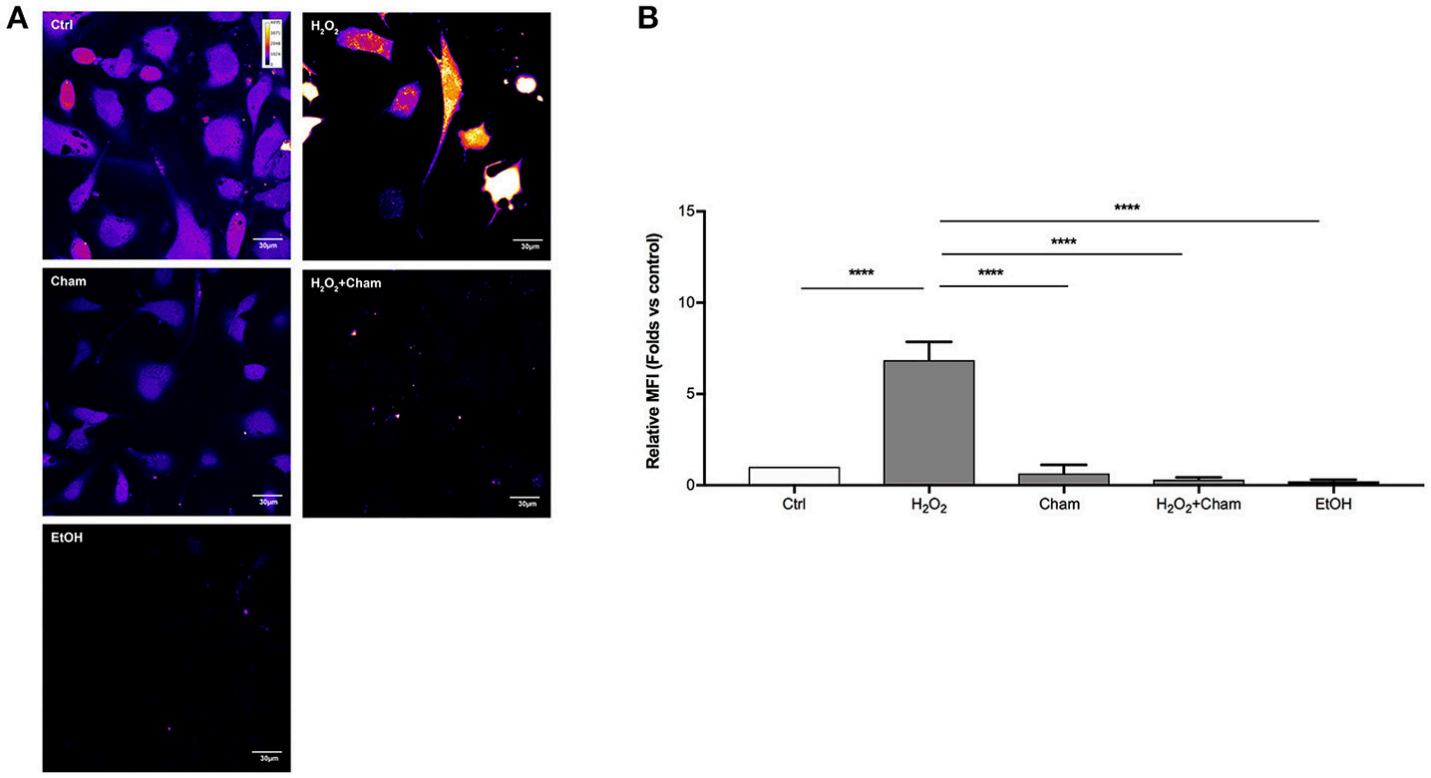
Chamazulene and ROS levels in confocal microscopy after H_2_O_2_ treatment. **(A)** Images obtained with confocal microscopy and DCFH-DA probe (magnification 60x). Fluorescence intensity is represented in pseudo color scale (“fire” in ImageJ software): H_2_O_2_ treatment for 3h induce augmented ROS levels as underlined by the changing color of cells compared to control and it was balanced by the simultaneous treatment with Cham. **(B)** Histograms illustrate the relative MFI toward control derived from images analysis and show high ROS levels when cells are treated with H_2_O_2_ and their decrease in a simultaneous treatment with Cham and H_2_O_2_. Ethanol does not affect ROS level. Data shown are mean ± SD of three independent experiments (H_2_O_2_: 6.86 ± 1.00; Cham: 0.65 ± 0.47; H_2_O_2_+Cham: 0.30 ± 0.13; EtOH: 0.22 ± 0.09); ^****^P < 0.0001.

**Figure 6:**
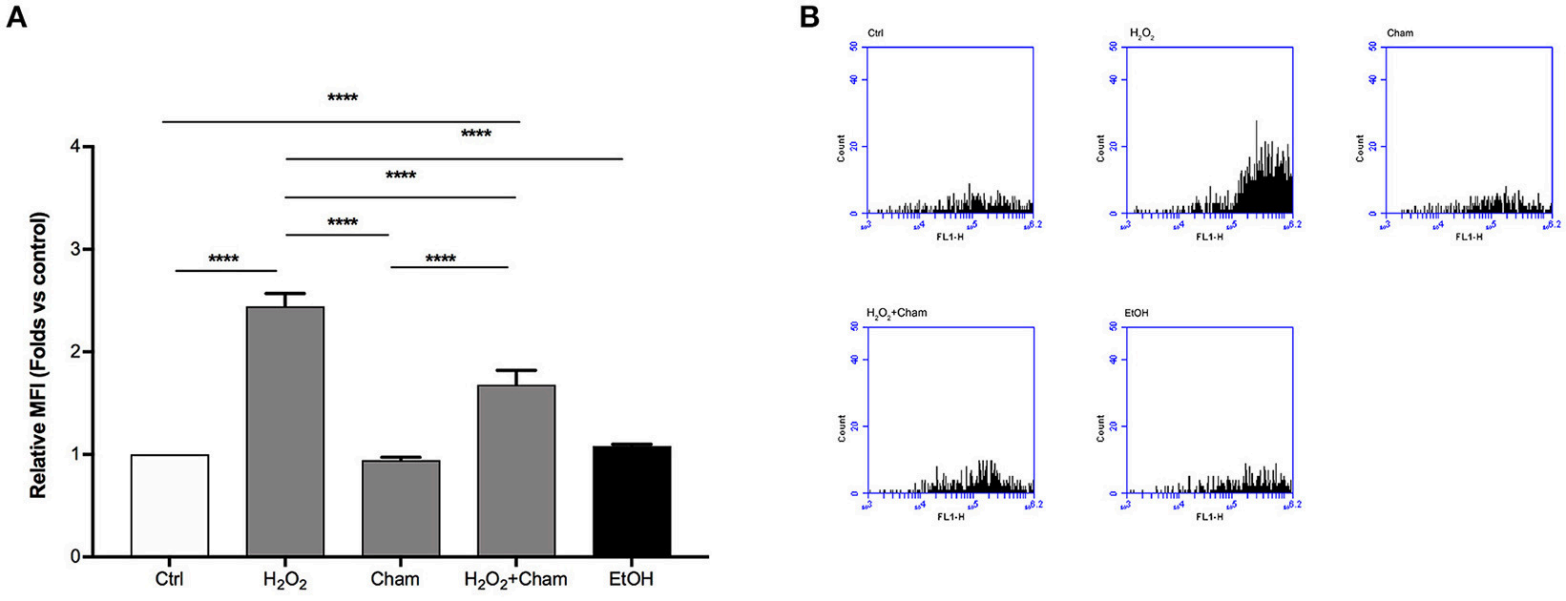
Chamazulene and ROS levels in flow cytometry after H_2_O_2_ treatment. **(A)** Histograms illustrate the relative MFI toward control derived from flow cytometry and show high ROS levels when cells are treated with H_2_O_2_ and their decrease in a simultaneous treatment with Cham and H_2_O_2_. Cham effect is not affected by ethanol. Data shown are mean ± SD of three independent experiments (H_2_O_2_: 2.44 ± 0.13; Cham: 0.94 ± 0.03; H_2_O_2_+Cham: 1.68 ± 0.14; EtOH: 1.08 ± 0.02); ^****^P < 0.0001 **(B)** Representative graphs of flow cytometry in which is illustrated the variation in fluorescence in different treatments.

ROS levels, due to H_2_O_2_ treatment for 3h, and their possible reduction induced by Cham, were quantified in a second set of experiments with confocal microscopy and flow cytometry using DCFH-DA probe. In confocal experiments, as shown in Figures 5A,B, BAECs stressed with H_2_O_2_ for 3 h revealed a higher production of ROS and this condition was reverted by simultaneous treatment with Cham 25 μg/ml. As shown, treatment with ethanol in which Cham was solubilized confirmed that ethanol had no effect on ROS production, not even Cham 25 μg/ml added alone to the culture medium had any effect on ROS levels. These results were confirmed by flow cytometry experiments (Figures 6A,B).

## Discussion

Cells are continuously exposed to physical and chemical stressors. When endogenous and exogenous defenses are not sufficient to balance the production of free radicals, ROS and RNS, cells enter in an oxidative stress status which can be deleterious for the structure and function of important molecules like nucleic acids, proteins and lipids (Birben et al., 2012). Several pathological conditions can contribute to the definition of such status, characterized by the rise of reactive molecules; as an example, endothelial cells exposure to high concentrations of glucose, as it occurs in diabetic disease, can increase ROS production, enhancing endothelial dysfunction that characterizes this condition (Rahimi et al., 2005). In this scenario, the role of antioxidant molecules endogenously produced in cells or derived from exogenous sources, represents the first line of investigation in order to assess any increased cell defense against oxidative stress status and thus preventing further cell damage. Scavenger activity of exogenous antioxidants is usually tested primarily with chemical assays, like the ABTS assay (Floegel et al., 2011). Then, in order to classify a molecule as an antioxidant in complex systems such as living cells, it is important to evaluate how the molecule behaves in a cell model of oxidative stress status. A molecule recently studied for its capability to scavenge free radicals is Cham (Capuzzo et al., 2014), a sesquiterpene spontaneously derived from matricine and present in high concentrations in chamomile (*Matricaria chamomilla* L.) essential oil. Previous studies on Cham underline its antioxidant activity as the molecule is able to scavenge preformed free radicals (Capuzzo et al., 2014; Formisano et al., 2015). Our aim, in this research paper, was to evaluate for the first time the role of Cham as an antioxidant in endothelial cells in two different models of oxidative stress. Since data showing the effect of Cham in cultured cells are still unavailable, our first line of investigation aimed to the determination of the concentration of the molecule by which treating cells. Results obtained on BAECs treated with different concentrations of Cham (Figure 2) showed no toxicity of the molecule. These data were used to calculate the EC_50_ of Cham at 3 h, 25 μg/ml, and it was used as our starting point to study any antioxidant effect of the molecule on cells. Cham was tested in two acute stress model that induced rise in ROS: cells were treated with HG or H_2_O_2_ for 3 h (Zhou et al., 2015; Nadeev et al., 2016). ROS levels were assessed using two different approaches: confocal microscopy and flow cytometry. Results obtained in confocal microscopy analysis showed a reduction in ROS levels when cells were simultaneously treated with HG or H_2_O_2_ and Cham (Figures 3, 5), and these data were confirmed by flow cytometry analysis (Figures 4, 6). These experiments underline the effect of Cham respect to different stimuli, high glucose and H_2_O_2_. High glucose was chosen to mimic the hyperglycemic state characteristic of diabetes. In this condition endothelial oxidative stress occurs thorough many pathways, including formation of peroxynitrite, reduced NO production, inactivation and/or reduction of expression of antioxidant enzymes, formation of AGEs (advanced glycation end products; Incalza et al., 2018). H_2_O_2_ represents a direct stronger insult that is able to induce covalent modifications of cysteine thiolate residues located in active and allosteric sites of specific proteins resulting in alterations on their activity and function. Moreover, high concentration of H_2_O_2_ evokes inflammatory responses leading to growth arrest and ultimately cell death (Sies, 2017).

Therefore, in these models ROS levels rise in different ways, but, in both conditions, Chamazulene was able to balance them. The novelty of this study can be pointed out, as done before, underlining that it has been the first work in which antioxidant activity of Cham has been tested on a cell model, even if, as previously said, results obtained were linked to short treatment times, reflecting only acute effects and not long term events such as gene expression. Furthermore, it could be interesting to evaluate how Cham acts in a cell model increasing the exposure time to different stressors and evaluating the effect on gene transcription. Another aspect that can be interesting to investigate, essential for the complete determination of its antioxidant activity, could be the determination of the kinetics and the dinamics of Chamazulene in *in vivo* studies.

Many natural antioxidants counteract oxidative stress, and their use leads to an improvement in ROS generation-associated diseases. New substances with antioxidant properties to balance ROS overproduction and favor NO bioavailability can be developed with the aim of preventing oxidative stress-induced vascular damage, and Cham could be included in the list of these molecules. Further studies on Cham and its potential application will reinforce the efficacy of natural beneficial nutritional components in delaying the onset of vascular dysfunction and maintaining or restoring vascular health.

## Author contributions

CB, RC, MG, and RL conceived the study, assisted its design and revised the manuscript for important intellectual content. GQ, RL, FF, and SA carried out the experiments, statistical analysis, and interpreted the results with the other authors. GQ wrote the manuscript. All authors read, edited and approved the final manuscript.

### Conflict of interest statement

The authors declare that the research was conducted in the absence of any commercial or financial relationships that could be construed as a potential conflict of interest.
